# Diamine oxidase supplementation improves symptoms in patients with histamine intolerance

**DOI:** 10.1007/s10068-019-00627-3

**Published:** 2019-05-24

**Authors:** Wolfgang J. Schnedl, Michael Schenk, Sonja Lackner, Dietmar Enko, Harald Mangge, Florian Forster

**Affiliations:** 1 Practice for General Internal Medicine, Dr. Theodor Körnerstrasse 19b, 8600 Bruck/Mur, Austria; 2 Das Kinderwunsch Institut Schenk GmbH, Am Sendergrund 11, 8143 Dobl, Austria; 3 Immunology and Pathophysiology, Otto Loewi Research Centre, Medical University of Graz, Heinrichstrasse 31a, 8010 Graz, Austria; 4 Institute of Laboratory Medicine, General Hospital Steyr, Sierninger Straße 170, 4400 Steyr, Austria; 5 Clinical Institute of Medical and Chemical Laboratory Diagnosis, Medical University of Graz, Auenbruggerplatz 30, 8036 Graz, Austria; 6 Sciotec Diagnostic Technology GmbH, Ziegelfeldstrasse 3, 3430 Tulln, Austria

**Keywords:** Histamine, Diamine oxidase, Malabsorption, Gastrointestinal, Extra-intestinal

## Abstract

Histamine intolerance (HIT) is thought to be caused by a disproportionate amount of histamine in the body. The enzyme diamine oxidase (DAO) is considered for the gastrointestinal degradation of histamine. For this open-label interventional pilot study, we identified 28 patients with HIT. For 4 weeks, they were instructed to take DAO capsules before meals. Then, throughout a follow-up period, they were instructed not to take the DAO. We used a questionnaire that included 22 symptoms, which were divided into 4 categories, as well as a symptom severity score. All symptoms improved significantly during the oral supplementation of DAO. During the follow-up period, without DAO supplementation, the symptoms sum scores increased again. The symptom intensity score was reduced for all symptoms. We have demonstrated, a significant reduction of every HIT-related symptom and its intensity due to DAO oral supplements.

The ClinicalTrials.gov identifier (NCT number) is NCT03298568.

## Introduction

Biogenic amines including histamine are produced by bacterial decarboxylation in food (Doeun et al., 2017). If the amount of ingested biogenic amines is high and/or their degradation is inhibited or disturbed in the body, then histamine is thought to cause multiple gastrointestinal (GI) symptoms. These may be accompanied by extra-intestinal symptoms including cardiovascular, respiratory and skin complaints (Reese et al., 2017). Symptoms of a disproportionate amount of histamine are thought to be caused by the reduced activity of the enzyme diamine oxidase (DAO). The clinical diagnosis of HIT is challenging. Although, patients with low serum DAO values, two or more GI symptoms described for HIT, and a reduction of complaints due to a histamine-reduced diet may be diagnosed with HIT. However, DAO is considered the main extracellular enzyme for the intestinal degradation of histamine and other biogenic amines (Elmore et al., 2007; Jones and Kearns, 2011). Therefore, the oral supplementation of commercially available DAO food supplements, prepared from pig kidney, has been suggested as a treatment of histamine intolerance (Smolinska et al., 2014). With this study, we aimed to demonstrate that the oral DAO supplementation improves symptoms, including GI, cardiovascular, respiratory and skin complaints in patients suffering from HIT.

## Materials and methods

For this open-label interventional pilot study, we identified, in an analysis of outpatients’ charts, 56 patients with recurring functional, nonspecific abdominal complaints and serum DAO values < 10 U/mL. At the time of diagnosis of HIT, all of these patients received written information on histamine intolerance and the histamine-reduced diet. A registered dietician helped develop an individually tailored diet to ensure nutritional adequacy. All of included patients maintained the histamine-reduced diet, although diet compliance was not separately evaluated for this study. Every patient had an easing of symptoms with the histamine-reduced diet. Although, they experienced a recurrence of functional nonspecific abdominal complaints, with serum DAO < 10 U/mL, at two separate evaluations, within 6 months before start of the study. Fifty-six patients were contacted by phone, 35 of them agreed to participate in the study. Thirty patients fulfilled the inclusion criteria with recurring functional nonspecific abdominal complaints and the screening DAO value < 10 U/mL, within 1 week before the study. Two patients were excluded during the study. One due to hospitalization and antibiotic therapy, and the other due to failing compliance. Therefore, 28 patients (male/female 7/21, median age 47.5 years, age range 19–72) completed the study according to the protocol and were included in the evaluation.

### Study design

The patients were instructed not to change their diets or medication throughout the study period of 8 weeks. Symptoms, compliance of DAO capsule ingestion, and determinations of serum DAO and histamine in plasma were recorded at each visit, in 2-week intervals. For 4 weeks the patients were instructed to take DAOSIN^®^ capsules, each containing 4.2 mg extracted pig kidney proteins with 0.3 mg DAO, before meals, up to three times per day. During the follow-up period of 4 weeks, the patients were instructed not to take DAO capsules. DAOSIN^®^ was provided by Sciotec Diagnostic Technologies, Tulln in Austria (DAOSIN^®^).

We used a standardized questionnaire to assess the symptoms experienced by patients before and during the study period. The questionnaire was based on known symptoms and the four histamine receptors (Schnedl et al., 2019). Twenty-two symptoms were listed in four categories: GI, cardiovascular, respiratory and skin complaints. For each symptom, a severity score, from 0 (no symptoms) to 5 (very intense), was used. The patients were instructed to fill out this questionnaire at each visit. A radio extraction assay DAO Rea 100 (Sciotec Diagnostic Technologies, Tulln, Austria) was used for determination of DAO in the serum. The amount of histamine in the plasma was measured with an enzyme linked immunoassay, Histamin ELISA BA 10-1000 (Diagnostika Nord GmbH & Co. KG, Nordhorn, Germany).

### Evaluation of other food intolerance/malabsorption

All patients were examined and had neither lactose intolerance, fructose malabsorption, *Helicobacter pylori* infection nor celiac disease. We used hydrogen (H_2_) breath tests for exclusion of lactose intolerance and fructose malabsorption (Gastrolyzer, Bedfont Scientific Inc., Kent, England). Either histologic evaluation of gastric mucosa or an enzyme-linked IgA immunosorbent assay (ELISA, Serion, Würzburg, Germany) showed absence of *Helicobacter pylori* infection. For screening of celiac disease antibodies against tissue transglutaminase were determined with anti-tTG IgA ELISA (Euro Diagnostica AB, Malmö, Sweden). The patients > 50 years old presented no pathology during a colonoscopy and the routine laboratory values were within normal range throughout the study.

### Statistics

At every visit, during the study, the symptoms score was evaluated. For the assessment of the overall symptom severity, the score’ sum was calculated. For detailed information on the distribution of symptoms, four categories—gastrointestinal, cardiovascular, respiratory and skin symptoms—were defined. Since the symptom score data is ordinally scaled, non-parametric tests were used for statistical calculations. The change in symptoms over time was calculated using the Friedman test. The Dunn’s multiple comparison test was used as a post hoc test to identify the significance of the changes in symptoms at each visit. The Wilcoxon signed-rank test was employed to compare the symptom score at the baseline (V1), after DAO ingestion (V3) and after the follow-up period (V5). It was also used to compare V1 and V5. Scatter-plots were used with medians and interquartile ranges (IQR), including minimal and maximal values (95% confidence interval). Calculations and graphs were done with GraphPad Prism Version 5.04.

## Results and discussion

**Fig. 1:**
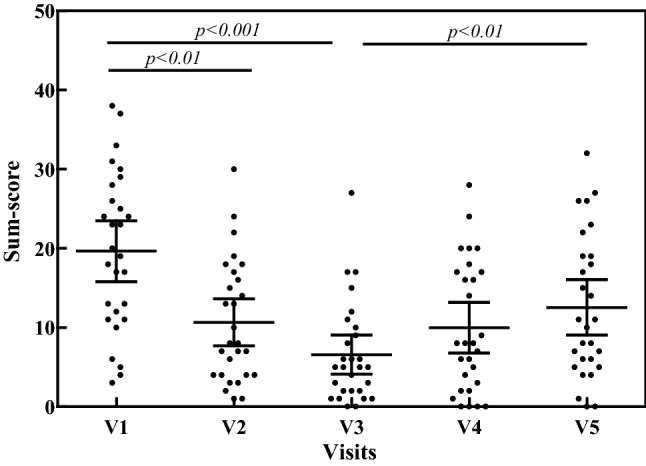
Scatter-plot of symptoms sum-score of all 22 HIT-related symptoms throughout the study period of 8 weeks. Oral supplementation of DAO from visit 1 to visit 3 (V1 to V3); no oral DAO from visit 3 to visit 5 (V3 to V5)

All 22 symptoms, including GI, cardiovascular, respiratory and skin complaints improved significantly (Table 1) during the oral supplementation of DAO from visit 1 to visit 3 (Wilcoxon *p *< 0.0001; Dunn *p *< 0.001). During the follow-up period from visit 3 to visit 5, the symptoms sum score increased (Wilcoxon *p *= 0.0008; Dunn *p *< 0.01), but, at visit 5, the symptoms sum score was still significantly lower than at visit 1 (Wilcoxon *p *= 0.0004) (Fig. 1).

**Table 1:** Mean symptoms sum-score (± SD) of all symptoms, 10 GI symptoms, the 5 most diagnostic GI symptoms, 4 cardiovascular symptoms, 4 respiratory symptoms and 4 skin complaints during the study period of 8 weeks

Symptoms	4 weeks of DAO supplementation		4 weeks without DAO		
	Visit 1 Mean (± SD)	Visit 2 Mean (± SD)	Visit 3 Mean (± SD)	Visit 4 Mean (± SD)	Visit 5 Mean (± SD)
All symptoms	19.6 (± 9.9)	10.6 (± 7.7)	6.6 (± 6.4) *p *< 0.0001	10 (± 8.3)	12.5 (± 9) *p *= 0.002
GI	11.2 (± 7.5)	6.1 (± 5.2)	3.8 (± 4.4) *p *< 0.0001	6.5 (± 5.9)	7.1 (± 5.3) *p *= 0.0058
Diagnostic GI	8.4 (± 5.6)	4.8 (± 4.1)	3.1 (± 3.4) *p *< 0.0001	5 (± 5)	5.4 (± 4.3) *p *= 0.0312
Cardiovascular	3.6 (± 2.8)	1.9 (± 2.1)	1 (± 1.2) *p *< 0.0001	1.3 (± 1.7)	1.8 (± 2.2) *p *= 0.0783
Respiratory	2.4 (± 2.7)	1.6 (± 2)	1.2 (± 1.6) *p *< 0.0001	1.2 (± 1.7)	2.4 (± 2.6) *p *= 0.0029
Skin	2.4 (± 3.1)	1 (± 1.5)	0.5 (± 1) *p *< 0.0001	0.9 (± 1.6)	1.2 (± 2.2) *p *= 0.1289

The significance of the change in symptoms over time was calculated using the Friedman test

*DAO* diamine oxidase, *GI* Gastrointestinal

### Gastrointestinal symptoms

**Fig. 2:**
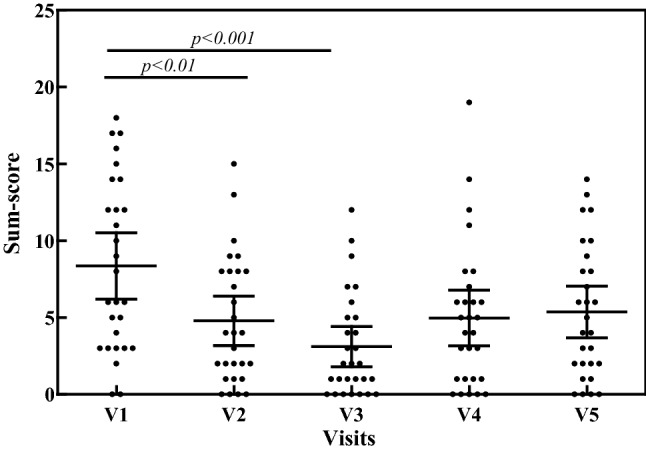
Scatter-plot of symptoms sum-score of the most common and diagnostic 5 GI symptoms (bloating, postprandial fullness, abdominal pain, belching and diarrhea) for HIT throughout the study period of 8 weeks. Oral supplementation of DAO from visit 1 to visit 3 (V1 to V3); no oral DAO from visit 3 to visit 5 (V3 to V5)

Evaluated GI symptoms, including abdominal pain, intestinal colic, bloating, diarrhea, constipation, nausea, belching, emesis, postprandial fullness and dysmenorrhea, significantly improved from visit 1 to visit 3 (Dunn *p *< 0.001; Wilcoxon *p *< 0.0001). During the follow-up period from visit 3 to visit 5, the symptoms sum score increased (Dunn *p *< 0.05; Wilcoxon *p *= 0.0044), but, at visit 5, the symptoms sum score was still significantly lower than visit 1 (Wilcoxon *p *= 0.0026). A significant reduction of symptoms with DAO ingestion was demonstrated using a sub-score, for the 5 most common and diagnostic GI symptoms of HIT: bloating, diarrhea, abdominal pain, belching and fullness. (Dunn *p *< 0.001; Wilcoxon *p *< 0.0001). During the 4 weeks of follow-up, a slight re-occurrence of symptoms was indicated (Wilcoxon *p *= 0.0086). Although, at visit 5, these symptoms were still significantly lower, compared to visit 1 (Wilcoxon *p *= 0.0059) (Fig. 2).

### Other symptoms of histamine intolerance

**Fig. 3:**
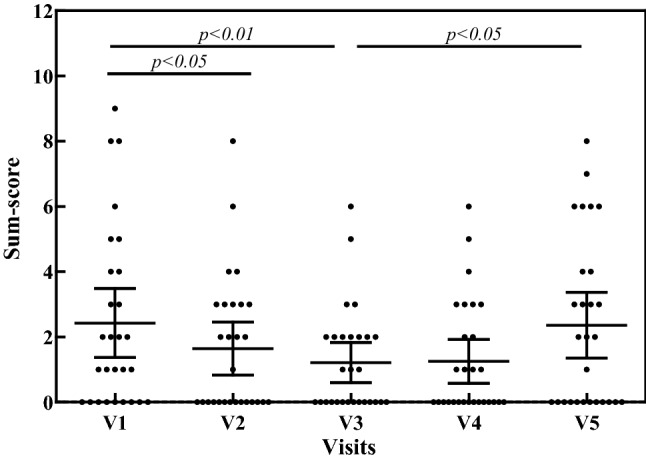
Scatter-plot of symptoms sum-score of respiratory symptoms (rhinorrhea, nose congestion, sneezing and asthma) throughout the study period of 8 weeks. Oral supplementation of DAO from visit 1 to visit 3 (V1 to V3); no oral DAO from visit 3 to visit 5 (V3 to V5)

Cardiovascular symptoms, including headache, vertigo, palpitations and collapse, significantly improved with DAO ingestion (Wilcoxon *p *< 0.0001). During the follow-up period from visit 3 to visit 5, the symptoms sum scores slightly increased (Wilcoxon *p *= 0.028), but, at visit 5, the symptoms were still significantly lower, when compared to visit 1 (Wilcoxon *p *= 0.0033). Respiratory symptoms, including rhinorrhea, nose congestion, sneezing and asthma, significantly improved from visit 1 to visit 3 (Dunn *p *< 0.01; Wilcoxon *p *= 0.0142). During the follow-up period from visit 3 to visit 5, the symptoms sum score increased (Dunn *p *< 0.05; Wilcoxon *p *= 0.007) and reached the sum score of visit 1 (Wilcoxon *p *= 0.9099) (Fig. 3). The symptoms of the skin—pruritus, urticaria, flush and swollen, reddened eyelids—significantly improved with DAO ingestion (Dunn *p *< 0.01; Wilcoxon *p *= 0.001). During the follow-up period from visit 3 to visit 5, the symptoms sum scores slightly increased (Wilcoxon *p *= 0.0236), but, at visit 5, the symptoms sum score, when compared to visit 1, was significantly lower (Wilcoxon *p *= 0.0364).

### Symptom intensity

**Fig. 4:**
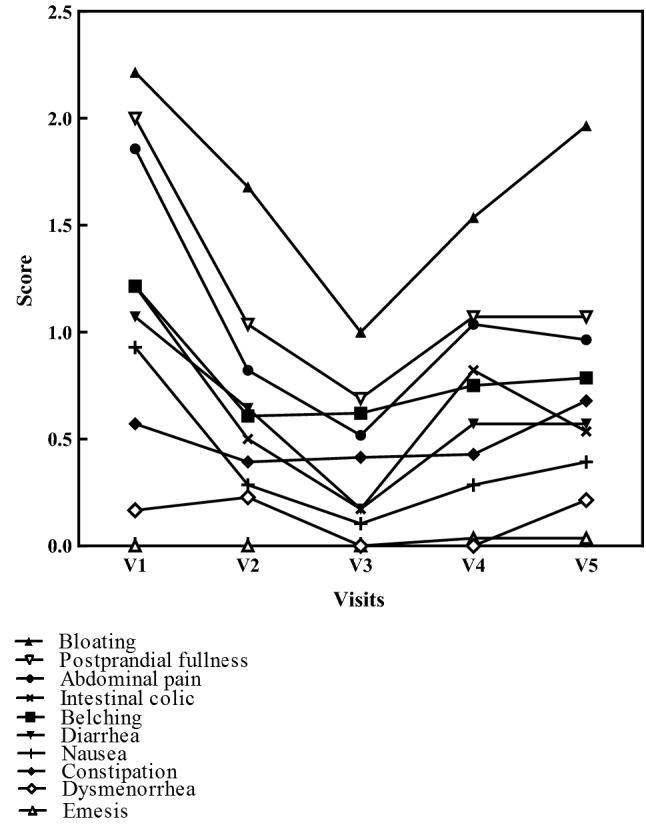
Mean intensity of GI symptoms (abdominal pain, intestinal colic, bloating, diarrhea, constipation, nausea, belching, emesis, postprandial fullness and dysmenorrhea) throughout the study period of 8 weeks. Oral supplementation of DAO from visit 1 to visit 3 (V1 to V3); no oral DAO from visit 3 to visit 5 (V3 to V5)

The symptom intensity, for every symptom a severity score from 0 (no symptoms) to 5 (very intense), indicated by patients was reduced for all symptoms—GI, cardiovascular, respiratory and skin complaints—due to the ingestion of DAO, as shown in Fig. 3. After the follow up period, none of the scores for symptom intensity returned to the intensity score of visit 1 (Fig. 4).

**Fig. 5:**
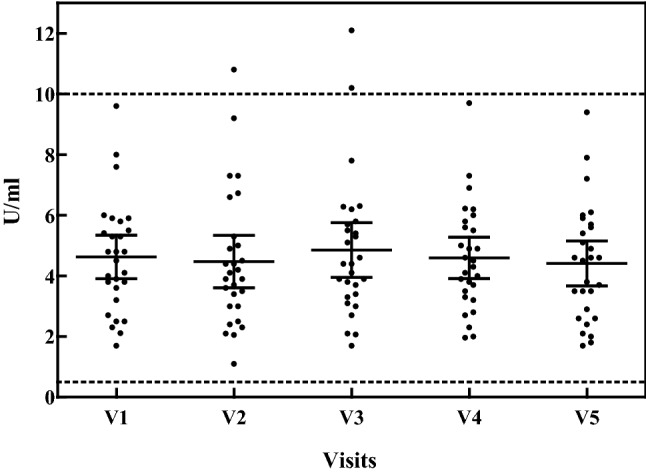
Scatter-plot of serum DAO values throughout the study period of 8 weeks. Oral supplementation of DAO from visit 1 to visit 3 (V1 to V3); no oral DAO from visit 3 to visit 5 (V3 to V5). Normal range for DAO in serum 0.5–10 U/mL

During DAO ingestion, 60.7% of patients showed slightly increased serum DAO values (Wilcoxon *p *= 0.2918), and DAO decreased again during the follow-up period (Wilcoxon *p *= 0.346) (Fig. 5). Histamine values, in plasma, remained unchanged during the study period.

### Diamine oxidase and histamine intolerance

Biogenic amines like histamine, putrescine, cadaverine, and agmatine are produced by bacterial decarboxylation in foods. Usually, ingesting of low amounts of biogenic amines does not affect general wellbeing. The amount of biogenic amines in food is affected by several factors, including: the manufacturing process, hygiene of raw materials, microbial composition, and the duration of fermentation (Doeun et al., 2017). If ingested food contains a high amount of biogenic amines and/or their degradation is inhibited or disturbed, histamine accumulates in the body (Reese et al., 2017). DAO is, with its ability to degrade biogenic amines, considered the main enzyme in GI and extracellular degradation of histamine (Elmore et al., 2007; Jones and Kearns, 2011). It is synthesized by apical enterocytes located in the intestinal villi and is released from the mucosa for digestion and into the blood circulation (Wollin et al., 1988). DAO from the white pea (*Lathyrus sativus*) reportedly modulates histamine toxicity in vitro. A combination of DAO and catalase protected against histamine toxicity and prevented H_2_O_2_-induced damage on the human intestinal Caco-2 cell line occurring during histamine’s oxidative deamination (Jumarie et al., 2017). An immune-regulatory influence of histamine within the gastrointestinal tract is known, but the effect of histamine on gut pathology, including inflammatory processes, is still poorly defined (Smolinska et al., 2014).

### Diamine oxidase in serum

Recent findings speculated that low serum DAO values may be responsible for the symptoms of HIT (Kacik et al., 2018). Although, serum DAO values have not been established to reflect gastrointestinal DAO activity. However, the diagnosis of HIT, with the presence of two or more functional non-specific GI symptoms, may be supported with measurements of DAO values in serum (Reese et al., 2017). In this study, for diagnosis of HIT, besides functional nonspecific abdominal complaints, serum DAO values < 10 U/mL were used. Throughout the study, we have demonstrated slightly increasing serum DAO values, due to oral DAO supplementation.

A histamine-reduced diet was effectively shown to improve symptoms and potentially elevate serum DAO, after approximately 2 months. Depending on the compliance with a histamine-reduced diet, an improvement of HIT-related symptoms was demonstrated in nearly 80% of patients. Additionally, symptom improvement, combined with an increase of serum DAO values, occurred in more than 50% of patients (Lackner et al., 2019). Patients described GI symptoms to be the most prominent and severe of the HIT-related symptoms, along with a variability of symptom combinations (Doeun et al., 2017). It was reported that DAO capsules, taken in combination with histamine-containing tea, reduced symptoms of HIT (Komericki et al., 2011). Recently, oral DAO supplementation was reported as effective in patients with headaches. It significantly reduced the duration of migraine attacks (Izquierdo-Casas et al., 2019) and, in another study, caused symptom relief in patients with urticaria (Yacoub et al., 2018). We have demonstrated a significant reduction of all HIT-related symptoms with the oral supplementation of DAO. Before this study, 79% of the included patients complained of postprandial fullness and, 68% had bloating and abdominal pain. After 4 weeks of DAO ingestion, these symptoms were significantly reduced to 50% bloating, 43% postprandial fullness and 25% abdominal pain. Interestingly, we recognized that symptoms at the end of the study did not reach the same level of severity as at the beginning. We speculate that a slight recovery of the intestinal mucosa, due to the ingestion of DAO may be responsible. At visit 5, only the respiratory symptoms reached the same symptom sum scores as at the beginning of the study. This, we attributed to the fall season and its increased number of colds. However, a placebo effect, influencing the improvement of symptoms, in this study cannot be ruled out (Eisenbruch and Enck, 2015). Additional studies, including placebo-controlled randomized trials with high numbers of patients and long treatment periods, are needed to fully evaluate oral DAO supplementation.

Generally, a nutritionally adequate histamine-reduced diet can be developed, based on the individual’s symptomatology, by reducing the amount of ingested histamine and biogenic amines. A histamine-reduced diet may present a challenge to HIT patients, because the composition of biogenic amines and levels of histamine in food and drinks are frequently unknown. In general, at least roughly 20 percent of patients with food intolerance/malabsorption cannot comply a comprehensive diet plan (Wilder-Smith et al., 2017). Therefore, we conducted this study in HIT patients and found that oral DAO supplementation helps reduce symptoms. We speculate that this may be due to its ability to degrade ingested intestinal histamine.

In conclusion, we have demonstrated that oral supplementation of the enzyme DAO before meals could help improve HIT-related symptoms and symptom intensity in HIT.
